# Supplementary material for: The Impact of COVID-19 and Related Public Health Measures on Hepatitis C Testing in Ontario, Canada
Source: Viruses. 2025 Aug 26;17(9):1163. doi: 10.3390/v17091163 (PMC12474368; doi:10.3390/v17091163)
Supplement: Supplementary file 1 [file viruses-17-01163-s001.zip › viruses-3790458-supplementary.pdf]

THE IMPACT OF COVID-19 AND RELATED PUBLIC HEALTH MEASURES ON HEPATITIS C TESTING IN ONTARIO, CANADA

SUPPLEMENT

Table S1. Selected ICES Datasets used in this study 3

Table S2. Case definition of history of homelessness, considering a two-year lookback period 4

Table S3. Algorithm to identify people who inject drugs 5

Table S4. Codes to identify cirrhosis 9

Table S5. Codes to identify decompensated cirrhosis 10

Table S6. Codes to identify hepatocellular carcinoma 12

Table S7. Codes used to identify liver transplantation 13

Table S8. Characteristics of the study cohort with HCV antibody test at index date 14

Table S9. Characteristics of the study cohort with HCV RNA test at index date 16

Table S10. Number of individuals per month with HCV antibody or RNA test, overall and by subgroups 18

Table S11. Results of interrupted time series analysis, overall and by priority populations. Outcome: either HCV antibody or RNA test 20

Table S12. Results of interrupted time series analysis, overall and by priority populations. Outcome: HCV antibody test 23

Table S13. Results of interrupted time series analysis, overall and by priority populations. Outcome: HCV RNA test 26

Figure S1. Observed HCV antibody test or RNA test (any result) counts during pre-pandemic and pandemic months 29

Figure S2. Study flowchart 30

Figure S3. Observed monthly rate of individuals per 1,000 with AB or RNA tests during pre-pandemic and pandemic periods, and the projected counterfactual pre-pandemic trend with 95% confidence band stratified by sex. 31

Figure S4. Observed monthly rate of individuals per 1,000 with AB or RNA tests during pre-pandemic and pandemic periods, and the projected counterfactual pre-pandemic trend with 95% confidence band stratified by immigration status. 32

Figure S5. Observed monthly rate of individuals per 1,000 with AB or RNA tests during pre-pandemic and pandemic periods, and the projected counterfactual pre-pandemic trend with 95% confidence band stratified by birth cohort. 33

Figure S6. Observed monthly rate of individuals per 1,000 with AB or RNA tests during pre-pandemic and pandemic periods, and the projected counterfactual pre-pandemic trend with 95% confidence band stratified by HIV status. 34

Figure S7. Observed monthly rate of individuals per 1,000 with AB or RNA tests during pre-pandemic and pandemic periods, and the projected counterfactual pre-pandemic trend with 95% confidence band stratified by homelessness status. 35

Figure S8. Observed monthly rate of individuals per 1,000 with AB or RNA tests during pre-pandemic and pandemic periods, and the projected counterfactual pre-pandemic trend with 95% confidence band stratified by people who inject drug status. 36

References 37

Table S1. Selected ICES Datasets used in this study

List of datasets	Full name	Description
ACG	The Johns Hopkins ACG® System Version 10	Used in combination with OHIP, DAD, NACRS, and SDS to calculate Adjusted Clinical Groups (ACGs), Resource Utilization Band (RUB), and frailty
CIC/IRCC	Immigration Refugees and Citizenship Canada, Permanent Residents database	Used to determine immigration status
CORR	Canadian Organ Replacement Registry	Used to identify individuals with liver transplantation
DAD	Discharge Abstract Database	Used to identify priority populations
DIN	The DRUGLIST file (DIN: Drug Identification Numbers)	Used with ODB to identify priority populations and to identify hepatitis C treatments
HIV	Human immunodeficiency virus	Used to determine HIV status
NACRS	National Ambulatory Care Reporting System	Used to identify priority populations
NMS	Narcotics Monitoring System	Used to identify priority populations
OCR	Ontario Cancer Registry	Used to identify individuals with hepatocellular carcinoma
ODB	Ontario Drug Benefit Claims	Used to identify priority populations and to identify hepatitis C treatments
OHIP	Ontario Health Insurance Plan Claims Database	Used to determine eligibility, date of last contact with health care system
OLIS	Ontario Laboratory Information System	Used to determine those with hepatitis C testing testing
OMHRS	Ontario Mental Health Reporting System	Used to identify priority populations
ON-MARG	Ontario Marginalization Index	Used to create the Age and labour force quintiles, Material resource quintiles, Racialized and newcomer populations quintile, and Households and dwellings quintile
PCCF	Postal Code Conversion File	Used to determine postal code for Ontario residency
POP	Ontario Population Estimates and Projections	Used to estimate the denominators for testing rates
RPDB	Registered Persons Database	Demographics information on birth date, sex, and death date<br>Used to estimate the denominators for testing rates
SDS	Same Day Surgery Database	Used to identify priority populations

Source: For more information, please check https://datadictionary.ices.on.ca/Applications/DataDictionary/Default.aspx

Table S2. Case definition of history of homelessness, considering a two-year lookback period (1, 2)

Database	Variable name	Indicator value	Description
**DAD**	DX10CODE1 to DXCODE25	“Z590” or “Z591”	ICD-10 Diagnostic Codes for “Homelessness” and “Inadequate housing”
	CMGDIAG	“Z590” or “Z591”	ICD-10 Diagnostic Codes for “Homelessness” and “Inadequate housing”
	PSTLCODE	“XX”; list of postal codes uniquely identifying homeless shelters	Used to indicate transient/homeless patients
**NACRS**	DX10CODE1 to DX10CODE10	“Z590” or “Z591”	ICD-10 Diagnostic Codes for “Homelessness” and “Inadequate housing”
	RESTYEP	“3” or “4”	Residence Type = “Homeless” or “Shelter”
	PSTLCODE	“XX”; uniquely identifying shelter code	Used to indicate transient/homeless patients
**OMHRS**	PREDX10CODE to PREDX10CODE11	“Z590” or “Z591”	ICD-10 Diagnostic Codes for “Homelessness” and “Inadequate housing”
	POSTDX10CODE1 to POSTDX10CODE24	“Z590” or “Z591”	ICD-10 Diagnostic Codes for “Homelessness” and “Inadequate housing”
	PRIOR_RESIDENCE	“6”	Prior residental status = “Homeless (with or without shelter)”
	USUAL_RESIDENCE	“8”	Usual residental status = “Homeless (with or without shelter)”
	ADMITFROM	“8”	Admitted from = “Homeless (with or without shelter)”
	DISCHLIVING	“8”	Living arrangemenet at discharge = “Homeless (with or without shelter)”
	PSTLCODE	“XX”; uniquely identifying shelter code	Used to indicate transient/homeless patients
**RPDB PSTLYEAR**		uniquely identifying shelter code	Used to indicate transient/homeless patients

Table S3. Algorithm to identify people who inject drugs (3)

Variable	Data source	Code type	Codes	Description
**Physician billing codes for drug use**				
**Drug dependence**	OHIP	DXCODE	304	Drug dependence
	OHIP	FEECODE	A680	Substance use – initial assessment
	OHIP	FEECODE	C680	Substance use – initial assessment
	OHIP	FEECODE	K680	Substance abuse – extended assessment
	OHIP	FEECODE	A957	Focused Practice Assessment – Addiction medicine
**Emergency department and hospitalization codes for drug use (DAD, NACRS, OMHRS)**				
**Opioid use**	DAD, NACRS	ICD-9	3040	Drug dependence: Opioid type dependence
	DAD, NACRS	ICD-9	3047	Drug dependence: Combinations of opioid type drug with any other
	DAD, NACRS	ICD-9	3055	Nondependent abuse of drugs: Opioid abuse
	DAD, NACRS	ICD-9	9650	Poisoning by analgesics, antipyretics, and antirheumatics: opiates and related narcotics
	DAD, NACRS	ICD-9	E8500	Accidental poisoning by heroin
	DAD, NACRS	ICD-10	F11	Mental and behavioural disorders due to use of opioids
	DAD, NACRS	ICD-10	R781	Finding of opiate drug in blood
	DAD, NACRS	ICD-10	T400	Poisoning by Opium
	DAD, NACRS	ICD-10	T401	Poisoning by Heroin
	DAD, NACRS	ICD-10	T402	Poisoning by Other opioids
	DAD, NACRS	ICD-10	T403	Poisoning by Methadone
	DAD, NACRS	ICD-10	T404	Poisoning by Other synthetic narcotics
	DAD, NACRS	ICD-10	T406	Poisoning by Other and unspecified narcotics
	OHMRS	DSM-IV	30400	Opioid Dependence
	OHMRS	DSM-IV	30550	Opioid abuse
**Cocaine use**	DAD, NACRS	ICD-9	3042	Drug dependence: Cocaine dependence
	DAD, NACRS	ICD-9	3056	Nondependent abuse of drugs: Cocaine abuse
	DAD, NACRS	ICD-9	970	Poisoning by central nervous system stimulants
	DAD, NACRS	ICD-10	F14	Mental and behavioural disorders due to use of cocaine
	DAD, NACRS	ICD-10	T405	Poisoning by: Cocaine
	DAD, NACRS	ICD-10	R782	Finding of cocaine in blood
	OHMRS	DSM-IV	30420	Cocaine dependence
	OHMRS	DSM-IV	30560	Cocaine abuse
**Amphetamines**	DAD, NACRS	ICD-9	3044	Drug dependence: Amphetamine and other psychostimulant dependence

Variable	Data source	Code type	Codes	Description
	DAD, NACRS	ICD-9	3057	Nondependent abuse of drugs: Amphetamine or related acting sympathomimetic abuse
	DAD, NACRS	ICD-9	9697	Poisoning by Psychostimulants
	DAD, NACRS	ICD-10	F15	Mental and behavioural disorders due to use of other stimulants, including caffeine
	DAD, NACRS	ICD-10	T436	Poisoning by psychotropic drugs, not elsewhere classified
	OHMRS	DSM-IV	30440	Amphetamine dependence
	OHMRS	DSM-IV	30570	Amphetamine abuse
**Sedatives**	DAD, NACRS	ICD-9	3041	Drug dependence: Sedative, hypnotic or anxiolytic dependence
	DAD, NACRS	ICD-9	9694	Poisoning by Benzodiazepine-based tranquilizers
	DAD, NACRS	ICD-9	3054	Nondependent abuse of drugs: Sedative, hypnotic or anxiolytic abuse
	DAD, NACRS	ICD-10	F13	Mental and behavioural disorders due to use of sedatives or hypnotics
	DAD, NACRS	ICD-10	T423	Poisoning by Barbiturates
	DAD, NACRS	ICD-10	T424	Poisoning by Benzodiazepines
	DAD, NACRS	ICD-10	T425	Poisoning by Mixed antiepileptics, not elsewhere classified
	DAD, NACRS	ICD-10	T426	Poisoning by Benzodiazepines
	DAD, NACRS	ICD-10	T427	Poisoning by Antiepileptic and sedative-hypnotic drugs, unspecified
	DAD, NACRS	ICD-10	T428	Poisoning by Antiparkinsonism drugs and other central muscle-tone depressants
	OHMRS	DSM-IV	30410	Sedative, hypnotic or anxiolytic dependence
	OHMRS	DSM-IV	30540	Sedative, hypnotic or anxiolytic abuse
**Other**	DAD, NACRS	ICD-9	292	Psychoses: Drug-induced mental disorders
	DAD, NACRS	ICD-9	3045	Drug dependence: Hallucinogen dependence
	DAD, NACRS	ICD-9	3046	Drug dependence: Other specified drug dependence
	DAD, NACRS	ICD-9	3048	Combinations of drug dependence excluding opioid type drug
	DAD, NACRS	ICD-9	3049	Drug dependence: Unspecified drug dependence
	DAD, NACRS	ICD-9	3053	Nondependent abuse of drugs: Hallucinogen abuse
	DAD, NACRS	ICD-9	3059	Nondependent abuse of drugs: Other, mixed, or unspecified drug abuse
	DAD, NACRS	ICD-9	6483	Other current conditions in the mother classifiable elsewhere, but complicating pregnancy, childbirth, or the puerperium: drug dependence
	DAD, NACRS	ICD-9	7960	Nonspecific abnormal toxicological findings
	DAD, NACRS	ICD-9	9621	Poisoning by Androgens and anabolic congeners
	DAD, NACRS	ICD-9	9658	Poisoning by Other specified analgesics and antipyretics
	DAD, NACRS	ICD-9	9663	Poisoning by Other and unspecified anticonvulsants including barbituates

Variable	Data source	Code type	Codes	Description
	DAD, NACRS	ICD-9	9664	Poisoning by Anti-Parkinsonism drugs
	DAD, NACRS	ICD-9	9670	Poisoning by Barbiturates
	DAD, NACRS	ICD-9	9684	Poisoning by Other and unspecified general anesthetics
	DAD, NACRS	ICD-9	9685	Poisoning by Surface [topical] and infiltration anesthetics
	DAD, NACRS	ICD-9	9696	Poisoning by Psychodysleptics [hallucinogens]
	DAD, NACRS	ICD-9	9698	Poisoning by Other specified psychotropic agents
	DAD, NACRS	ICD-9	9699	Poisoning by Unspecified psychotropic agent
	DAD, NACRS	ICD-9	970	Poisoning by central nervous system stimulants
	DAD, NACRS	ICD-10	F16	Mental and behavioural disorders due to use of hallucinogens
	DAD, NACRS	ICD-10	F19	Mental and behavioural disorders: multiple drug use and other psychoactive substances
	DAD, NACRS	ICD-10	T387	Poisoning by Androgens and anabolic congeners
	DAD, NACRS	ICD-10	T408	Poisoning by narcotics and psychodysleptics: Lysergide (LSD)
	DAD, NACRS	ICD-10	T409	Poisoning by Other and unspecified psychodysleptics [hallucinogens]
	DAD, NACRS	ICD-10	T412	Poisoning by Other and unspecified general anaesthetics
	DAD, NACRS	ICD-10	T438	Poisoning by Other psychotropic drugs, not elsewhere classified
	DAD, NACRS	ICD-10	T439	Poisoning by Psychotropic drug, unspecified
	DAD, NACRS	ICD-10	T507	Poisoning by Analeptics and opioid receptor antagonists
	DAD, NACRS	ICD-10	Z715	Drug abuse counselling and surveillance
	DAD, NACRS	ICD-10	Z503	Drug rehabilitation
	DAD, NACRS	ICD-10	Z722	Problems related to lifestyle - Drug use
	DAD, NACRS	ICD-10	Z8641	Personal history of psychoactive substance abuse
	OHMRS	DSM-IV	30450	Hallucinogen dependence
	OHMRS	DSM-IV	30480	Polysubstance dependence
	OHMRS	DSM-IV	30490	Other (or unknown) substance dependence
	OHMRS	DSM-IV	30530	Hallucinogen abuse
	OHMRS	DSM-IV	30590	Other (or unknown) substance abuse
**Opioid agonist treatment (OAT)**				
**Physician billing**	OHIP	FEE CODE	K682	OAT monthly management fee, intensive
	OHIP	FEE CODE	K683	OAT monthly management fee, maintenance
	OHIP	FEE CODE	K684	OAT monthly management fee, team premium
**Methadone**	ODB, NMS	PIN	02244290	Metadol-D
	ODB, NMS	PIN	02394596	Methadose

Variable	Data source	Code type	Codes	Description
	ODB, NMS	PIN	02394618	Methadose
	ODB, NMS	PIN	02481979	Methadone HCL concentrate
	ODB, NMS	PIN	02495872	Odan-methadone
	ODB, NMS	PIN	02495880	Odan-methadone
	ODB, NMS	PIN	09850619	Methadone
	ODB, NMS	PIN	09857499	Methadone compounding MMT
	ODB, NMS	PIN	02495783	Methadone HCL
**Buprenorphine/ Naloxone**	ODB, NMS	PIN	02408090	Mylan-buprenorph/naloxone
	ODB, NMS	PIN	02408104	Mylan-buprenorph/naloxone
	ODB, NMS	PIN	02424851	Teva-buprenorphine-naloxone
	ODB, NMS	PIN	02424878	Teva-buprenorphine-naloxone
	ODB, NMS	PIN	02453908	Act buprenorphine/naloxone
	ODB, NMS	PIN	02453916	Act buprenorphine/naloxone
	ODB, NMS	PIN	02502313	Buprenorphine HCL & naloxone HCL
	ODB, NMS	PIN	02502321	Buprenorphine HCL & naloxone HCL
	ODB, NMS	PIN	02502348	Buprenorphine HCL & naloxone HCL
	ODB, NMS	PIN	02502356	Buprenorphine HCL & naloxone HCL
	ODB, NMS	PIN	02502356	Buprenorphine
	ODB, NMS	PIN	02502356	Buprenorphine
	ODB, NMS	PIN	02502356	Buprenorphine
	ODB, NMS	PIN	02468085	Suboxone
	ODB, NMS	PIN	02468093	Suboxone
	ODB, NMS	PIN	02295695	Suboxone
	ODB, NMS	PIN	02295709	Suboxone
**Buprenorphine**	ODB, NMS	PIN	02474921	Probuphine
	ODB, NMS	PIN	02483084	Sublocade
	ODB, NMS	PIN	02483092	Sublocade

Table S4. Codes to identify cirrhosis

Definition: At least 2 physician visit (OHIP) or at least 1 hospitalization (DAD) or at least 1 ambulatory encounter (any NACRS source) with diagnostic code (excluding suspected diagnosis). NOTE: This is a validated algorithm.

**Code(s)**	**Description**
**ICD-9 codes**	All DXTYPEs are captured from all types of DAD/SDS/NACRS visits (e.g. scheduled admissions, NACRS source = any, ED visits that led to hospitalization, ED visits not seen by physician) unless otherwise specified
4561	ESOPH VARICES W/O BLEED
5712	ALCOHOL CIRRHOSIS LIVER
5715	CIRRHOSIS OF LIVER NOS
**ICD-10-CA codes**	All DXTYPEs are captured from all types of DAD/SDS/NACRS visits (e.g. scheduled admissions, NACRS source = any, ED visits that led to hospitalization, ED visits not seen by physician) unless otherwise specified
I859	Oesophageal varices without bleeding
I982	Oesophageal varices without bleeding in diseases classified elsewhere
I9820	Oesophageal varices in diseases classified elsewhere with bleeding
I9821	Oesophageal varices in diseases classified elsewhere without bleeding
K717	Toxic liver disease with fibrosis and cirrhosis of liver
K703	Alcoholic cirrhosis of liver
K746	Other and unspecified cirrhosis of liver
**OHIP diagnosis codes**	All OHIP non-lab billings are included (e.g., all locations, all billing specialties) unless otherwise specified
571	Cirrhosis of the liver (e.g., alcoholic cirrhosis, biliary cirrhosis)

Table S5. Codes to identify decompensated cirrhosis

Definition: At least 1 physician visit (OHIP) AND at least 1 hospitalization (DAD) with diagnostic or procedure code (excluding suspected diagnosis). NOTE: This is a validated algorithm.

Code(s)	Description
**ICD-9 codes**	All DXTYPEs are captured from all types of DAD/SDS/NACRS visits (e.g. scheduled admissions, NACRS source = any, ED visits that led to hospitalization, ED visits not seen by physician) unless otherwise specified
4560	ESOPHAG VARICES W BLEED
4562	BLEED ESOPH VAR OTH DIS
45620	BLEED ESOPH VAR OTH DIS
45621	ESOPH VARICE OTH DIS NOS
5722	HEPATIC COMA
5723	PORTAL HYPERTENSION
5724	HEPATORENAL SYNDROME
7824	JAUNDICE NOS
7895	ASCITES
**ICD-10-CA codes**	All DXTYPEs are captured from all types of DAD/SDS/NACRS visits (e.g. scheduled admissions, NACRS source = any, ED visits that led to hospitalization, ED visits not seen by physician) unless otherwise specified
I850	Oesophageal varices with bleeding
I864	Gastric varices
I9820	Oesophageal varices in diseases classified elsewhere with bleeding
I983	Oesophageal varices with bleeding in disease classified elsewhere
K721	Chronic hepatic failure
K729	Hepatic failure, unspecified
K766	Portal hypertension
K767	Hepatorenal syndrome
R17	Unspecified jaundice
R18	Ascites
**CCP codes**	
1006	INSERTION OF SENGSTAKEN TUBE
6691	PERCUTANEOUS ABDOMINAL PARACENTESIS
**CCI codes**	
1NA13BAFA	Control of bleeding, esophagus using endoscopic per orifice approach and banding (varices)
1NA13BAX7	Control of bleeding, esophagus using endoscopic per orifice approach and chemical agent [e.g. ethanolamine, murrhate sodium, polidocanol, sclerosants, tetradecyl sulfate]
1NA13BABD	Control of bleeding, esophagus using endoscopic per orifice approach and balloon (or Sengstaken) tube tamponade
1KQ76GPNR	Bypass, abdominal veins NEC using percutaneous transluminal approach and stent [TIPS] bypass terminating in abdominal vein

1OT52HA	Drainage, abdominal cavity using percutaneous (needle) approach
1OT52HATS	Drainage, abdominal cavity using percutaneous (needle) approach and leaving drainage tube in situ
**OHIP FEE codes**	All OHIP non-lab billings are included (e.g., all locations, all billing specialties) unless otherwise specified
J057	TRANSJUGULAR INTRAHEPATIC PORTOSYSTEMIC SHUNT (TIPS)
Z591	ABD.PERITONEUM,OMENTUM-PARACENTESIS-THERAPEUT.DRAIN. SAMPLE

Table S6. Codes to identify hepatocellular carcinoma (4)

Inpatient diagnostic codes	ICD-9	ICD-10
Malignant neoplasm of liver	155.0	C22.9
Hepatocellular carcinoma		C22.0<br>81703
Combined hepatocellular and cholangiocarcinoma		81803
**Ontario Cancer Registry codes**		
Morphology		Topography
81703 (NOS)<br>81723 (scirrhous)<br>81733 (spindle)<br>81743 (clear cell)<br>81753 (pleomorphic)<br>81803 (Combined hepatocellular carcinoma and cholangiocarcinoma)		C220
**Death codes**		
Death registry main cause of death		Other Cause of Death (ICD-10)

Table S7. Codes used to identify liver transplantation

Definition: At least 1 hospitalization (DAD) OR 1 ambulatory encounter (any NACRS source) with transplant diagnostic or procedure code OR 1 physician billing with OHIP feecode OR transplant receipt in CORR.

Code(s)	Description
**ICD-9 codes**	All DXTYPEs are captured from all types of DAD/SDS/NACRS visits (e.g. scheduled admissions, NACRS source = any, ED visits that led to hospitalization, ED visits not seen by physician) unless otherwise specified
V427	LIVER TRANSPLANT STATUS
**ICD-10-CA codes**	All DXTYPEs are captured from all types of DAD/SDS/NACRS visits (e.g. scheduled admissions, NACRS source = any, ED visits that led to hospitalization, ED visits not seen by physician) unless otherwise specified
T86400	Liver transplant rejection
T86401	Liver transplant failure
T86402	Liver transplant infection
Z944	Liver transplant status
**CCP codes**	
624	LIVER TRANSPLANT
6240	LIVER TRANSPLANT
6241	AUXILIARY LIVER TRANSPLANT
6249	OTHER TRANSPLANT OF LIVER
**CCI codes**	
1OA85	Transplant, liver
1OA85LAXXK	Transplant, liver of a deceased donor full size liver
1OA85VCXXK	Transplant, liver of a deceased donor multi organ liver with intestine/ pancreas/spleen/stomach [or any combination of]
1OA85WLXXJ	Transplant, liver of a living donor split liver
1OA85WLXXK	Transplant, liver of a deceased donor split liver (or reduced ?paediatric÷ size liver)
**OHIP FEE codes**	All OHIP non-lab billings are included (e.g., all locations, all billing specialties) unless otherwise specified
S294	LIVER-EXC.-LIVER TRANSPLANT-RECIPIENT.
S295	DIGEST.SYST.-LIVER-REPEAT LIVER TRANSPLANT
G254	D&T,PROC.GASTROENTEROLOGY-MGM.POST LIVER TRANSPL'TIMMUNOSUPN
E765	LIVER-RECONST./REPAIR.HEPATIC ARTERY(RE-ANASTOM.CONDUIT)ADD

Table S8. Characteristics of the study cohort with HCV antibody test at index date

Characteristics	Pre-pandemic<br>01/01/2015 – 29/02/2020<br>N=2,337,727	Pandemic<br>01/03/2020 – 31/12/2022<br>N=1,302,963
**Birth year, n (%)**		
<1945	155,568 (6.7)	69,073 (5.3)
1945-1965	647,894 (27.7)	290,151 (22.3)
1965-1974	377,482 (16.1)	177,360 (13.6)
>1974	1,156,783 (49.5)	766,379 (58.8)
**Male sex, n (%)**	1,083,427 (46.3)	589,124 (45.2)
**Immigrant status, n (%)**	682,102 (29.2)	384,835 (29.5)
**Rural, n (%)**	156,960 (6.7)	93,667 (7.2)
**Neighborhood income quintile, n (%)**		
1st quintile (lowest)	526,962 (22.5)	292,790 (22.5)
2nd quintile	478,959 (20.5)	268,739 (20.6)
3rd quintile	456,703 (19.5)	256,874 (19.7)
4th quintile	436,103 (18.7)	243,371 (18.7)
5th quintile	430,955 (18.4)	236,777 (18.2)
**Household and dwellings quintile, n (%)**		
1st quintile (lowest)	506,228 (21.7)	274,438 (21.1)
2nd quintile	390,068 (16.7)	213,889 (16.4)
3rd quintile	372,768 (15.9)	208,291 (16.0)
4th quintile	400,263 (17.1)	229,198 (17.6)
5th quintile	646,319 (27.6)	363,170 (27.9)
**Material resources quintile, n (%)**		
1st quintile (lowest)	499,664 (21.4)	266,232 (20.4)
2nd quintile	463,980 (19.8)	262,918 (20.2)
3rd quintile	439,101 (18.8)	252,137 (19.4)
4th quintile	432,422 (18.5)	239,660 (18.4)
5th quintile	480,479 (20.6)	268,039 (20.6)
**Racialized and newcomer populations quintile, n (%)**		
1st quintile (lowest)	256,824 (11.0)	146,046 (11.2)
2nd quintile	302,229 (12.9)	174,060 (13.4)
3rd quintile	378,159 (16.2)	212,558 (16.3)
4th quintile	522,560 (22.4)	292,512 (22.4)
5th quintile	855,874 (36.6)	463,810 (35.6)
**Homelessness, n (%)**	6,745 (0.3)	6,220 (0.5)
**PWID, n (%) (ever)**	305,343 (13.1)	166,515 (12.8)
**HIV positivity, n (%)**	10,434 (0.4)	6,712 (0.5)
**ADG, mean (sd)**	6.11 (3.80)	6.10 (3.84)
**ADG categories, n (%)**		
0-3	652,601 (27.9)	371,756 (28.5)
4-8	1,103,471 (47.2)	604,186 (46.4)
9-10	268,319 (11.5)	148,978 (11.4)
>10	313,336 (13.4)	178,043 (13.7)
**Advanced liver disease**		
Cirrhosis, n (%)	32,637 (1.4)	19,480 (1.5)
Decompensated cirrhosis, n (%)	8,035 (0.3)	4,438 (0.3)
HCC, n (%)	2,131 (0.1)	1,127 (0.1)

Liver transplant recipients, n (%)	1,037 (0.0)	584 (0.0)

* This table includes anyone with any HCV antibody test, regardless of result. The characteristics are as of the index date, which is the earliest of the antibody or RNA or genotype test date, or antiviral initiation or SVR attainment in the study window. Those who tested multiple times within a given study period were counted only once. Those who were tested in both periods were counted once for each period, the same baseline characteristics applied.

ADG: aggregated diagnostic groups; HCC: hepatocellular carcinoma; HCV: hepatitis C virus; HIV: human immunodeficiency virus; N: number of observations; PWID: people who inject drugs; RNA: ribonucleic acid; sd: standard deviation

Table S9. Characteristics of the study cohort with HCV RNA test at index date

**Characteristics**	**Pre-pandemic 01/01/2015 – 29/02/2020 N= 66,254**	**Pandemic 01/03/2020 – 31/12/2022 N=34,560**
**Birth year, n (%)**		
<1945	3,019 (4.6%)	1,056 (3.1%)
1945-1965	30,122 (45.5%)	11,057 (32.0%)
1965-1974	12,291 (18.6%)	6,497 (18.8%)
>1974	20,822 (31.4%)	15,950 (46.2%)
**Male sex, n (%)**	39,940 (60.3%)	20,591 (59.6%)
**Immigrant status, n (%)**	10,238 (15.5%)	4,448 (12.9%)
**Rural, n (%)**	7,350 (11.1%)	4,546 (13.2%)
**Neighborhood income quintile, n (%)**		
1st quintile (lowest)	23,861 (36.0%)	13,292 (38.5%)
2nd quintile	14,578 (22.0%)	7,579 (21.9%)
3rd quintile	11,133 (16.8%)	5,596 (16.2%)
4th quintile	8,681 (13.1%)	4,242 (12.3%)
5th quintile	7,522 (11.4%)	3,504 (10.1%)
**Household and dwellings quintile, n (%)**		
1st quintile (lowest)	7,931 (12.0%)	3,373 (9.8%)
2nd quintile	8,115 (12.2%)	3,937 (11.4%)
3rd quintile	10,025 (15.1%)	4,951 (14.3%)
4th quintile	13,855 (20.9%)	7,561 (21.9%)
5th quintile	24,424 (36.9%)	13,284 (38.4%)
**Material resources quintile, n (%)**		
1st quintile (lowest)	8,548 (12.9%)	3,970 (11.5%)
2nd quintile	9,508 (14.4%)	4,667 (13.5%)
3rd quintile	10,825 (16.3%)	5,361 (15.5%)
4th quintile	13,478 (20.3%)	6,960 (20.1%)
5th quintile	21,991 (33.2%)	12,148 (35.2%)
**Racialized and newcomer populations quintile, n (%)**		
1st quintile (lowest)	11,464 (17.3%)	6,553 (19.0%)
2nd quintile	11,669 (17.6%)	6,448 (18.7%)
3rd quintile	12,095 (18.3%)	6,398 (18.5%)
4th quintile	13,401 (20.2%)	6,743 (19.5%)
5th quintile	15,721 (23.7%)	6,964 (20.2%)
**Homelessness, n (%)**	1,623 (2.4%)	1,829 (5.3%)
**PWID, n (%) (ever)**	35,874 (54.1%)	21,811 (63.1%)
**HIV positivity, n (%)**	1,852 (2.8%)	818 (2.4%)
**ADG, mean (sd)**	7.50 (4.26)	7.37 (4.27)
**ADG categories, n (%)**		
0-3	12,671 (19.1%)	7,037 (20.4%)
4-8	28,202 (42.6%)	14,494 (41.9%)
9-10	9,573 (14.4%)	4,952 (14.3%)
>10	15,808 (23.9%)	8,077 (23.4%)
**Advanced liver disease**		
Cirrhosis, n (%)	9,723 (14.7%)	2,839 (8.2%)
Decompensated cirrhosis, n (%)	2,141 (3.2%)	528 (1.5%)
HCC, n (%)	844 (1.3%)	211 (0.6%)

Liver transplant recipients, n (%)	472 (0.7%)	107 (0.3%)

* This table includes anyone with any HCV RNA test, regardless of result. The characteristics are as of the index date, which is the earliest of the antibody or RNA or genotype test date, or antiviral initiation or SVR attainment in the study window. Those who tested multiple times within a given study period were counted only once. Those who were tested in both periods were counted once for each period, the same baseline characteristics applied.

ADG: aggregated diagnostic groups; HCC: hepatocellular carcinoma; HCV: hepatitis C virus; HIV: human immunodeficiency virus; N: number of observations; PWID: people who inject drugs; RNA: ribonucleic acid; sd: standard deviation

Table S10. Number of individuals per month with HCV antibody or RNA test, overall and by subgroups

	Pre-pandemic 01/01/2015 – 29/02/2020		Pandemic 01/03/2020 – 31/12/2022	
**Either antibody or RNA test**	**N=2,353,170**		**N=1,312,942**	
	**Median**	**IQR**	**Median**	**IQR**
**Overall**	55,356	13,512	51,857	8,188
**Sex**				
F	29,175	6,998	27,793	4,162
M	26,330	6,242	23,778	3,694
**Birth cohort**				
<1945	3,546	436	2,648	404
1945-1964	14,634	3,772	11,398	1,556
1965-1974	8,811	1,364	7,059	931
≥1975	28,265	7,969	30,220	5,350
**PWID ever**				
No	46,465	11,913	43,727	7,394
Yes	8,900	1,919	7,830	873
**Immigrant status**				
No	39,230	10,176	36,553	5,599
Yes	16,406	3,526	15,262	2,853
**HIV status**				
No	54,632	13,531	51,323	8,053
Yes	694	113	489	106
**Homelessness**				
No	55,146	13,379	51,550	8,155
Yes	212	123	346	60
**Antibody test**	**N=2,337,727**		**N=1,302,963**	
**Overall**	53,421	13,816	50,760	7,991
**Sex**				
F	28,419	6,965	27,390	4,054
M	25,121	6,555	23,103	3,576
**Birth cohort**				
<1945	3,448	499	2,629	383
1945-1964	13,902	3,990	11,045	1,473
1965-1974	8,484	1,361	6,828	938
≥1975	27,811	7,829	29,746	5,299
**PWID ever**				
No	45,640	12,085	43,391	7,356
Yes	7,789	1,593	7,067	826

**Immigrant status**				
No	37,616	10,293	35,650	5,510
Yes	16,123	3,502	15,142	2,853
**HIV status**				
No	52,771	13,784	50,264	7,810
Yes	626	103	457	104
**Homelessness**				
No	53,244	13,692	50,514	7,973
Yes	174	96	268	48
**RNA test**	**N=66,254**		**N=34,560**	
**Overall**	2,473	415	1,634	228
**Sex**				
F	948	166	645	86
M	1,513	233	975	135
**Birth cohort**				
<1945	112	62	40	16
1945-1964	1,258	372	502	79
1965-1974	461	90	314	32
≥1975	684	291	766	154
**PWID ever**				
No	1,116	277	540	92
Yes	1,384	273	1,074	207
**Immigrant status**				
No	2,082	343	1,409	219
Yes	392	92	198	28
**HIV status**				
No	2,388	392	1,592	225
Yes	81	38	44	11
**Homelessness**				
No	2,426	443	1,525	208
Yes	52	36	102	29

HCV: hepatitis C virus; HIV: human immunodeficiency virus; IQR: interquartile range; N: number of observations. PWID: people who inject drugs; RNA: ribonucleic acid

Table S11. Results of interrupted time series analysis, overall and by priority populations. Outcome: either HCV antibody or RNA test

Model Parameter	Estimate	SE	p-value
**Overall**			
Intercept	3.268	0.162	0.000
Pre-pandemic testing rate slope, monthly	0.021	0.004	0.000
Study period (pandemic, ref: pre-pandemic)	-1.382	0.165	0.000
Pandemic testing rate slope, monthly	0.019	0.008	0.020
Difference in testing rate slopes during pandemic vs pre-pandemic periods	-0.001	0.010	0.896
**Female**			
Intercept	3.383	0.172	0.000
Pre-pandemic testing rate slope, monthly	0.022	0.004	0.000
Study period (pandemic, ref: pre-pandemic)	-1.315	0.180	0.000
Pandemic testing rate slope, monthly	0.017	0.009	0.062
Difference in testing rate slopes during pandemic vs pre-pandemic periods	-0.005	0.011	0.642
**Male**			
Intercept	3.149	0.154	0.000
Pre-pandemic testing rate slope, monthly	0.019	0.004	0.000
Study period (pandemic, ref: pre-pandemic)	-1.452	0.151	0.000
Pandemic testing rate slope, monthly	0.022	0.007	0.005
Difference in testing rate slopes during pandemic vs pre-pandemic periods	0.002	0.009	0.786
**<1945**			
Intercept	2.322	0.117	0.000
Pre-pandemic testing rate slope, monthly	0.013	0.003	0.000
Study period (pandemic, ref: pre-pandemic)	-0.514	0.104	0.000
Pandemic testing rate slope, monthly	0.006	0.005	0.258
Difference in testing rate slopes during pandemic vs pre-pandemic periods	-0.007	0.006	0.278
**1945-1964**			
Intercept	3.306	0.232	0.000
Pre-pandemic testing rate slope, monthly	0.029	0.006	0.000
Study period (pandemic, ref: pre-pandemic)	-1.848	0.303	0.000
Pandemic testing rate slope, monthly	0.006	0.014	0.661
Difference in testing rate slopes during pandemic vs pre-pandemic periods	-0.022	0.017	0.185
**1965-1974**			
Intercept	3.963	0.168	0.000
Pre-pandemic testing rate slope, monthly	0.016	0.004	0.000
Study period (pandemic, ref: pre-pandemic)	-1.847	0.200	0.000
Pandemic testing rate slope, monthly	0.021	0.009	0.032
Difference in testing rate slopes during pandemic vs pre-pandemic periods	0.005	0.011	0.686

Model Parameter	Estimate	SE	p-value
**1975+**			
Intercept	3.231	0.142	0.000
Pre-pandemic testing rate slope, monthly	0.020	0.003	0.000
Study period (pandemic, ref: pre-pandemic)	-1.202	0.130	0.000
Pandemic testing rate slope, monthly	0.023	0.007	0.001
Difference in testing rate slopes during pandemic vs pre-pandemic periods	0.004	0.008	0.648
**PWID (ever): YES**			
Intercept	8.798	0.403	0.000
Pre-pandemic testing rate slope, monthly	0.024	0.010	0.017
Study period (pandemic, ref: pre-pandemic)	-2.556	0.371	0.000
Pandemic testing rate slope, monthly	0.003	0.019	0.865
Difference in testing rate slopes during pandemic vs pre-pandemic periods	-0.021	0.023	0.368
**PWID (ever): NO**			
Intercept	2.897	0.147	0.000
Pre-pandemic testing rate slope, monthly	0.020	0.004	0.000
Study period (pandemic, ref: pre-pandemic)	-1.300	0.153	0.000
Pandemic testing rate slope, monthly	0.021	0.008	0.008
Difference in testing rate slopes during pandemic vs pre-pandemic periods	0.001	0.009	0.943
**Immigrant: YES**			
Intercept	5.397	0.191	0.000
Pre-pandemic testing rate slope, monthly	0.014	0.005	0.005
Study period (pandemic, ref: pre-pandemic)	-2.081	0.203	0.000
Pandemic testing rate slope, monthly	0.035	0.010	0.001
Difference in testing rate slopes during pandemic vs pre-pandemic periods	0.021	0.012	0.086
**Immigrant: NO**			
Intercept	2.782	0.155	0.000
Pre-pandemic testing rate slope, monthly	0.021	0.004	0.000
Study period (pandemic, ref: pre-pandemic)	-1.203	0.158	0.000
Pandemic testing rate slope, monthly	0.015	0.008	0.065
Difference in testing rate slopes during pandemic vs pre-pandemic periods	-0.006	0.009	0.496
**HIV: YES**			
Intercept	39.720	1.519	0.000
Pre-pandemic testing rate slope, monthly	-0.095	0.042	0.028
Study period (pandemic, ref: pre-pandemic)	-11.342	2.447	0.000
Pandemic testing rate slope, monthly	0.029	0.109	0.790
Difference in testing rate slopes during pandemic vs pre-pandemic periods	0.124	0.117	0.295
**HIV: NO**			
Intercept	3.220	0.161	0.000
Pre-pandemic testing rate slope, monthly	0.021	0.004	0.000
Study period (pandemic, ref: pre-pandemic)	-1.371	0.164	0.000
Pandemic testing rate slope, monthly	0.019	0.008	0.019
Difference in testing rate slopes during pandemic vs pre-pandemic periods	-0.001	0.010	0.885

Model Parameter	Estimate	SE	p-value
**Homelessness: YES**			
Intercept	19.435	1.335	0.000
Pre-pandemic testing rate slope, monthly	0.031	0.038	0.406
Study period (pandemic, ref: pre-pandemic)	-4.736	2.262	0.039
Pandemic testing rate slope, monthly	0.047	0.100	0.640
Difference in testing rate slopes during pandemic vs pre-pandemic periods	0.016	0.107	0.882
**Homelessness: NO**			
Intercept	3.261	0.162	0.000
Pre-pandemic testing rate slope, monthly	0.020	0.004	0.000
Study period (pandemic, ref: pre-pandemic)	-1.379	0.164	0.000
Pandemic testing rate slope, monthly	0.019	0.008	0.021
Difference in testing rate slopes during pandemic vs pre-pandemic periods	-0.001	0.010	0.902

HCV: hepatitis C virus; HIV: human immunodeficiency virus; PWID: people who inject drug; ref; reference; RNA: ribonucleic acid; SE: standard error

Table S12. Results of interrupted time series analysis, overall and by priority populations. Outcome: HCV antibody test

Model Parameter	Estimate	SE	p-value
**Overall**			
Intercept	3.134	0.159	0.000
Pre-pandemic testing rate slope, monthly	0.021	0.004	0.000
Study period (pandemic, ref: pre-pandemic)	-1.341	0.162	0.000
Pandemic testing rate slope, monthly	0.020	0.008	0.017
Difference in testing rate slopes during pandemic vs pre-pandemic periods	-0.001	0.010	0.891
**Female**			
Intercept	3.287	0.170	0.000
Pre-pandemic testing rate slope, monthly	0.022	0.004	0.000
Study period (pandemic, ref: pre-pandemic)	-1.283	0.178	0.000
Pandemic testing rate slope, monthly	0.017	0.009	0.055
Difference in testing rate slopes during pandemic vs pre-pandemic periods	-0.005	0.011	0.648
**Male**			
Intercept	2.977	0.150	0.000
Pre-pandemic testing rate slope, monthly	0.020	0.004	0.000
Study period (pandemic, ref: pre-pandemic)	-1.400	0.147	0.000
Pandemic testing rate slope, monthly	0.022	0.007	0.003
Difference in testing rate slopes during pandemic vs pre-pandemic periods	0.002	0.009	0.802
**<1945**			
Intercept	2.250	0.118	0.000
Pre-pandemic testing rate slope, monthly	0.013	0.003	0.000
Study period (pandemic, ref: pre-pandemic)	-0.494	0.104	0.000
Pandemic testing rate slope, monthly	0.006	0.005	0.234
Difference in testing rate slopes during pandemic vs pre-pandemic periods	-0.007	0.006	0.274
**1945-1964**			
Intercept	2.964	0.228	0.000
Pre-pandemic testing rate slope, monthly	0.031	0.006	0.000
Study period (pandemic, ref: pre-pandemic)	-1.770	0.300	0.000
Pandemic testing rate slope, monthly	0.007	0.014	0.603
Difference in testing rate slopes during pandemic vs pre-pandemic periods	-0.024	0.016	0.157
**1965-1974**			
Intercept	3.808	0.163	0.000
Pre-pandemic testing rate slope, monthly	0.016	0.004	0.000
Study period (pandemic, ref: pre-pandemic)	-1.780	0.196	0.000
Pandemic testing rate slope, monthly	0.021	0.009	0.027
Difference in testing rate slopes during pandemic vs pre-pandemic periods	0.005	0.011	0.636

Model Parameter	Estimate	SE	p-value
**1975+**			
Intercept	3.194	0.139	0.000
Pre-pandemic testing rate slope, monthly	0.019	0.003	0.000
Study period (pandemic, ref: pre-pandemic)	-1.177	0.128	0.000
Pandemic testing rate slope, monthly	0.023	0.006	0.001
Difference in testing rate slopes during pandemic vs pre-pandemic periods	0.004	0.008	0.601
**PWID (ever): YES**			
Intercept	7.794	0.348	0.000
Pre-pandemic testing rate slope, monthly	0.021	0.008	0.012
Study period (pandemic, ref: pre-pandemic)	-2.171	0.320	0.000
Pandemic testing rate slope, monthly	0.003	0.016	0.855
Difference in testing rate slopes during pandemic vs pre-pandemic periods	-0.019	0.020	0.348
**PWID (ever): NO**			
Intercept	2.821	0.146	0.000
Pre-pandemic testing rate slope, monthly	0.021	0.004	0.000
Study period (pandemic, ref: pre-pandemic)	-1.282	0.153	0.000
Pandemic testing rate slope, monthly	0.021	0.007	0.007
Difference in testing rate slopes during pandemic vs pre-pandemic periods	0.000	0.009	0.968
**Immigrant: YES**			
Intercept	5.273	0.188	0.000
Pre-pandemic testing rate slope, monthly	0.015	0.005	0.002
Study period (pandemic, ref: pre-pandemic)	-2.051	0.201	0.000
Pandemic testing rate slope, monthly	0.035	0.010	0.001
Difference in testing rate slopes during pandemic vs pre-pandemic periods	0.020	0.012	0.088
**Immigrant: NO**			
Intercept	2.646	0.152	0.000
Pre-pandemic testing rate slope, monthly	0.021	0.004	0.000
Study period (pandemic, ref: pre-pandemic)	-1.159	0.155	0.000
Pandemic testing rate slope, monthly	0.015	0.008	0.057
Difference in testing rate slopes during pandemic vs pre-pandemic periods	-0.006	0.009	0.491
**HIV: YES**			
Intercept	35.028	1.429	0.000
Pre-pandemic testing rate slope, monthly	-0.054	0.040	0.182
Study period (pandemic, ref: pre-pandemic)	-10.964	2.309	0.000
Pandemic testing rate slope, monthly	0.041	0.102	0.692
Difference in testing rate slopes during pandemic vs pre-pandemic periods	0.094	0.111	0.396
**HIV: NO**			
Intercept	3.092	0.158	0.000
Pre-pandemic testing rate slope, monthly	0.021	0.004	0.000
Study period (pandemic, ref: pre-pandemic)	-1.330	0.161	0.000
Pandemic testing rate slope, monthly	0.020	0.008	0.016
Difference in testing rate slopes during pandemic vs pre-pandemic periods	-0.001	0.010	0.882

Model Parameter	Estimate	SE	p-value
**Homelessness: YES**			
Intercept	16.838	1.095	0.000
Pre-pandemic testing rate slope, monthly	0.005	0.031	0.872
Study period (pandemic, ref: pre-pandemic)	-3.860	1.829	0.038
Pandemic testing rate slope, monthly	0.026	0.081	0.745
Difference in testing rate slopes during pandemic vs pre-pandemic periods	0.022	0.087	0.805
**Homelessness: NO**			
Intercept	3.128	0.159	0.000
Pre-pandemic testing rate slope, monthly	0.021	0.004	0.000
Study period (pandemic, ref: pre-pandemic)	-1.338	0.162	0.000
Pandemic testing rate slope, monthly	0.019	0.008	0.017
Difference in testing rate slopes during pandemic vs pre-pandemic periods	-0.001	0.010	0.896

HCV: hepatitis C virus; HIV: human immunodeficiency virus; PWID: people who inject drug; ref; reference; RNA: ribonucleic acid; SE: standard error

Table S13. Results of interrupted time series analysis, overall and by priority populations. Outcome: HCV RNA test

Model Parameter	Estimate	SE	p-value
**Overall**			
Intercept	0.179	0.008	0.000
Pre-pandemic testing rate slope, monthly	0.000	0.000	0.239
Study period (pandemic, ref: pre-pandemic)	-0.049	0.010	0.000
Pandemic testing rate slope, monthly	0.000	0.000	0.554
Difference in testing rate slopes during pandemic vs pre-pandemic periods	0.000	0.001	0.966
**Female**			
Intercept	0.131	0.006	0.000
Pre-pandemic testing rate slope, monthly	0.000	0.000	0.790
Study period (pandemic, ref: pre-pandemic)	-0.037	0.008	0.000
Pandemic testing rate slope, monthly	0.000	0.000	0.324
Difference in testing rate slopes during pandemic vs pre-pandemic periods	0.000	0.000	0.459
**Male**			
Intercept	0.228	0.010	0.000
Pre-pandemic testing rate slope, monthly	0.000	0.000	0.082
Study period (pandemic, ref: pre-pandemic)	-0.062	0.012	0.000
Pandemic testing rate slope, monthly	0.000	0.001	0.750
Difference in testing rate slopes during pandemic vs pre-pandemic periods	0.000	0.001	0.678
**<1945**			
Intercept	0.097	0.006	0.000
Pre-pandemic testing rate slope, monthly	0.000	0.000	0.022
Study period (pandemic, ref: pre-pandemic)	-0.016	0.009	0.076
Pandemic testing rate slope, monthly	-0.001	0.000	0.095
Difference in testing rate slopes during pandemic vs pre-pandemic periods	0.000	0.000	0.523
**1945-1964**			
Intercept	0.421	0.016	0.000
Pre-pandemic testing rate slope, monthly	-0.003	0.000	0.000
Study period (pandemic, ref: pre-pandemic)	-0.092	0.022	0.000
Pandemic testing rate slope, monthly	-0.001	0.001	0.318
Difference in testing rate slopes during pandemic vs pre-pandemic periods	0.002	0.001	0.170
**1965-1974**			
Intercept	0.209	0.011	0.000
Pre-pandemic testing rate slope, monthly	0.000	0.000	0.090
Study period (pandemic, ref: pre-pandemic)	-0.081	0.014	0.000
Pandemic testing rate slope, monthly	0.000	0.001	0.864
Difference in testing rate slopes during pandemic vs pre-pandemic periods	-0.001	0.001	0.418
**1975+**			
Intercept	0.062	0.004	0.000
Pre-pandemic testing rate slope, monthly	0.001	0.000	0.000

Model Parameter	Estimate	SE	p-value
Study period (pandemic, ref: pre-pandemic)	-0.029	0.005	0.000
Pandemic testing rate slope, monthly	0.000	0.000	0.632
Difference in testing rate slopes during pandemic vs pre-pandemic periods	-0.001	0.000	0.010
**PWID (ever): YES**			
Intercept	1.355	0.083	0.000
Pre-pandemic testing rate slope, monthly	0.003	0.002	0.166
Study period (pandemic, ref: pre-pandemic)	-0.451	0.096	0.000
Pandemic testing rate slope, monthly	0.000	0.005	0.962
Difference in testing rate slopes during pandemic vs pre-pandemic periods	-0.003	0.006	0.557
**PWID (ever): NO**			
Intercept	0.101	0.003	0.000
Pre-pandemic testing rate slope, monthly	-0.001	0.000	0.000
Study period (pandemic, ref: pre-pandemic)	-0.021	0.004	0.000
Pandemic testing rate slope, monthly	0.000	0.000	0.284
Difference in testing rate slopes during pandemic vs pre-pandemic periods	0.000	0.000	0.101
**Immigrant: YES**			
Intercept	0.168	0.007	0.000
Pre-pandemic testing rate slope, monthly	-0.001	0.000	0.000
Study period (pandemic, ref: pre-pandemic)	-0.042	0.009	0.000
Pandemic testing rate slope, monthly	0.000	0.000	0.556
Difference in testing rate slopes during pandemic vs pre-pandemic periods	0.001	0.001	0.177
**Immigrant: NO**			
Intercept	0.182	0.008	0.000
Pre-pandemic testing rate slope, monthly	0.000	0.000	0.732
Study period (pandemic, ref: pre-pandemic)	-0.051	0.010	0.000
Pandemic testing rate slope, monthly	0.000	0.000	0.585
Difference in testing rate slopes during pandemic vs pre-pandemic periods	0.000	0.001	0.739
**HIV: YES**			
Intercept	6.303	0.228	0.000
Pre-pandemic testing rate slope, monthly	-0.058	0.006	0.000
Study period (pandemic, ref: pre-pandemic)	-0.359	0.382	0.349
Pandemic testing rate slope, monthly	-0.010	0.017	0.564
Difference in testing rate slopes during pandemic vs pre-pandemic periods	0.048	0.018	0.009
**HIV: NO**			
Intercept	0.171	0.008	0.000
Pre-pandemic testing rate slope, monthly	0.000	0.000	0.393
Study period (pandemic, ref: pre-pandemic)	-0.049	0.009	0.000
Pandemic testing rate slope, monthly	0.000	0.000	0.566
Difference in testing rate slopes during pandemic vs pre-pandemic periods	0.000	0.001	0.879
**Homelessness: YES**			
Intercept	3.638	0.358	0.000
Pre-pandemic testing rate slope, monthly	0.035	0.010	0.001

Model Parameter	Estimate	SE	p-value
Study period (pandemic, ref: pre-pandemic)	-1.109	0.631	0.083
Pandemic testing rate slope, monthly	0.024	0.028	0.405
Difference in testing rate slopes during pandemic vs pre-pandemic periods	-0.011	0.030	0.704
**Homelessness: NO**			
Intercept	0.178	0.008	0.000
Pre-pandemic testing rate slope, monthly	0.000	0.000	0.117
Study period (pandemic, ref: pre-pandemic)	-0.048	0.010	0.000
Pandemic testing rate slope, monthly	0.000	0.000	0.474
Difference in testing rate slopes during pandemic vs pre-pandemic periods	0.000	0.001	0.996

HCV: hepatitis C virus; HIV: human immunodeficiency virus; PWID: people who inject drug; ref; reference; RNA: ribonucleic acid; SE: standard error

Figure S1. Observed HCV antibody test or RNA test (any result) counts during pre-pandemic and pandemic months

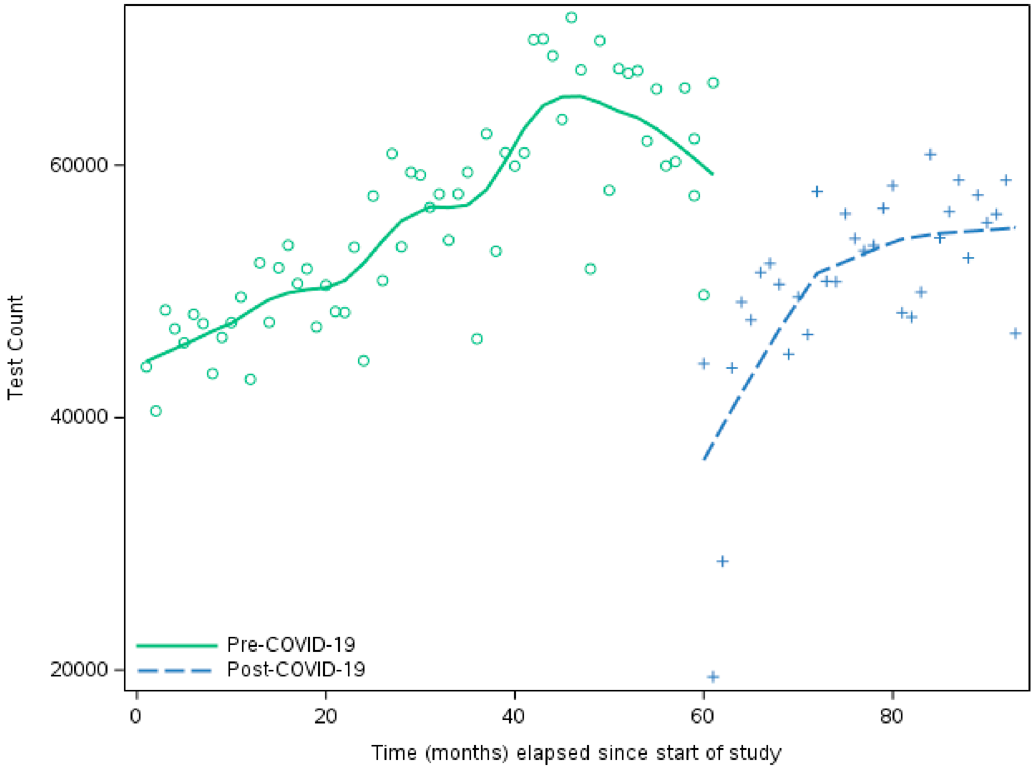

HCV: hepatitis C virus; RNA: ribonucleic acid

Figure S2. Study flowchart

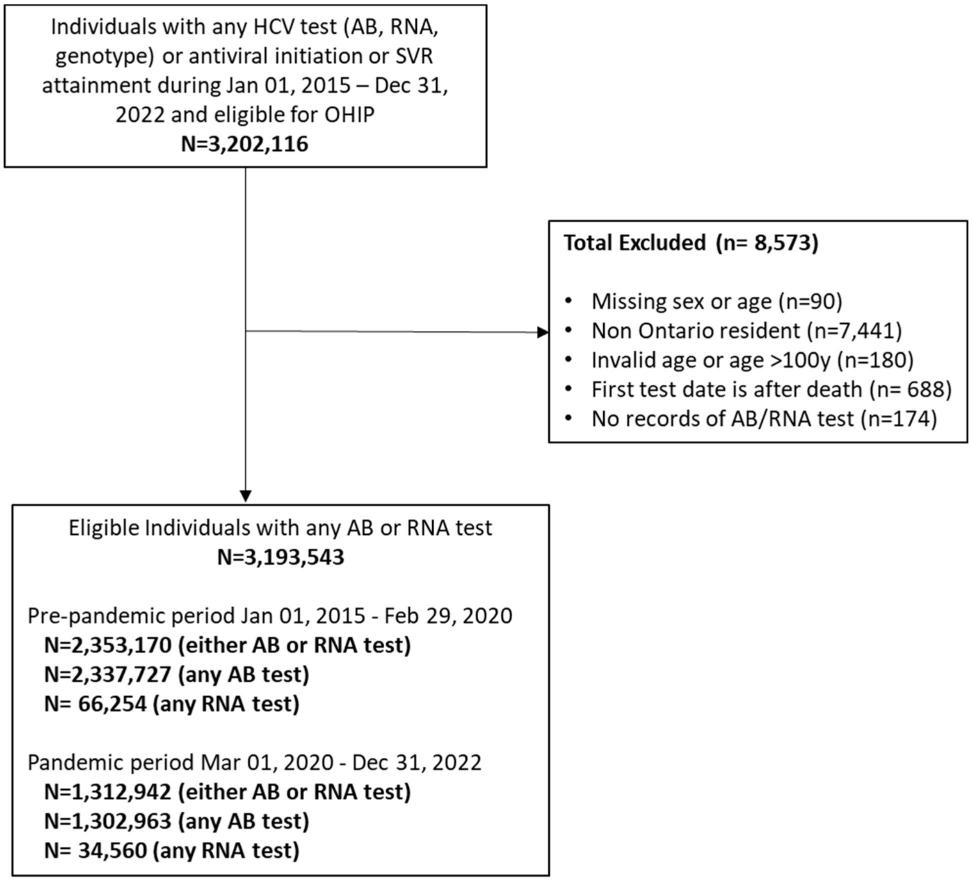

*Individuals who were tested in both periods, are counted once for each period.
AB; antibody; RNA: ribonucleic acid; OHIP: Ontario Health Insurance Plan; SVR: sustained viral response

Figure S3. Observed monthly rate of individuals per 1,000 with AB or RNA tests during pre-pandemic and pandemic periods, and the projected counterfactual pre-pandemic trend with 95% confidence band stratified by sex. AB: antibody; HCV: hepatitis C virus; RNA: ribonucleic acid.

Female

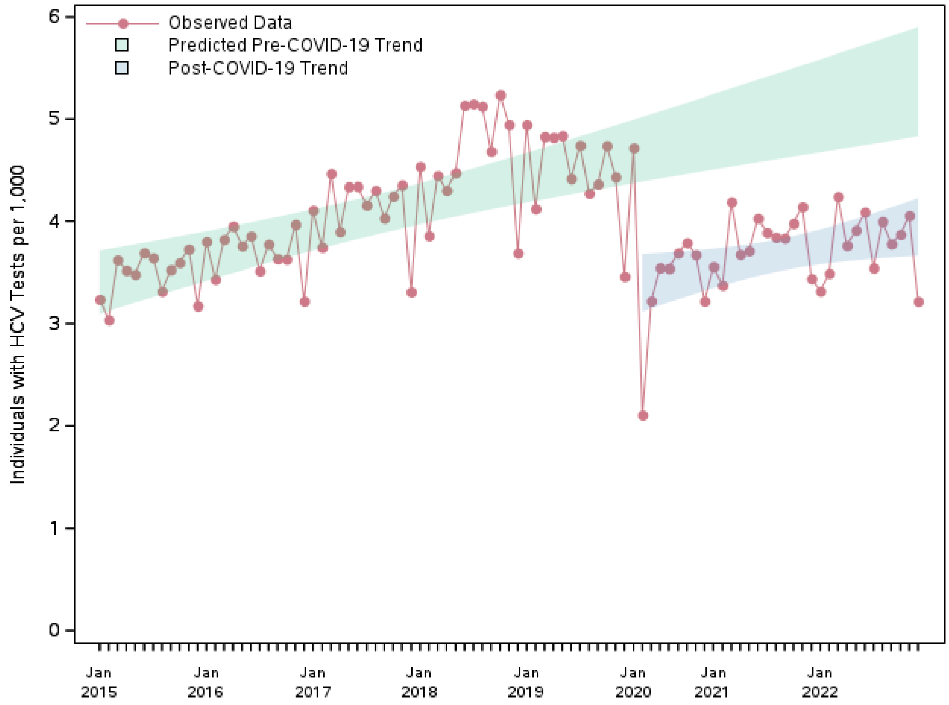

Male

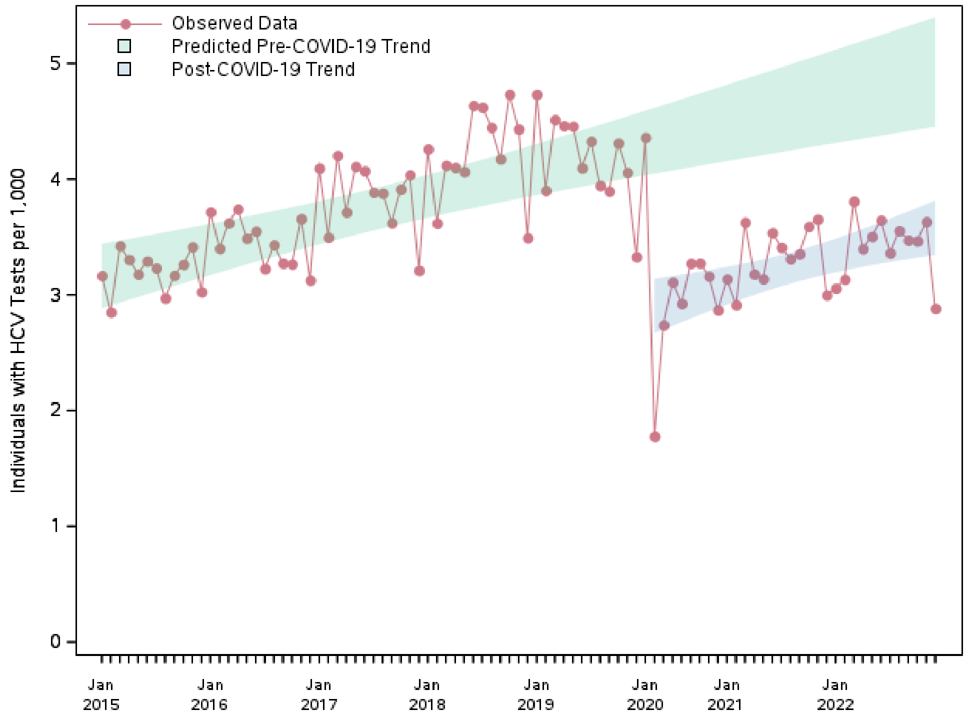

Figure S4. Observed monthly rate of individuals per 1,000 with AB or RNA tests during pre-pandemic and pandemic periods, and the projected counterfactual pre-pandemic trend with 95% confidence band stratified by immigration status. AB: antibody; HCV: hepatitis C virus; RNA: ribonucleic acid.

Immigration: YES

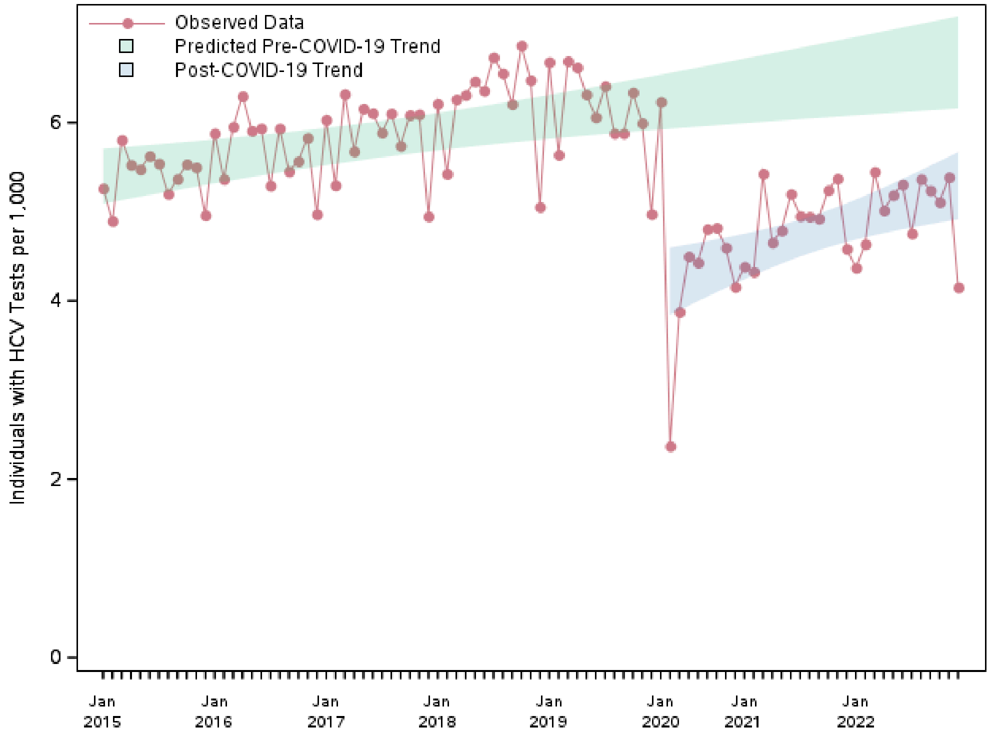

Immigration: NO

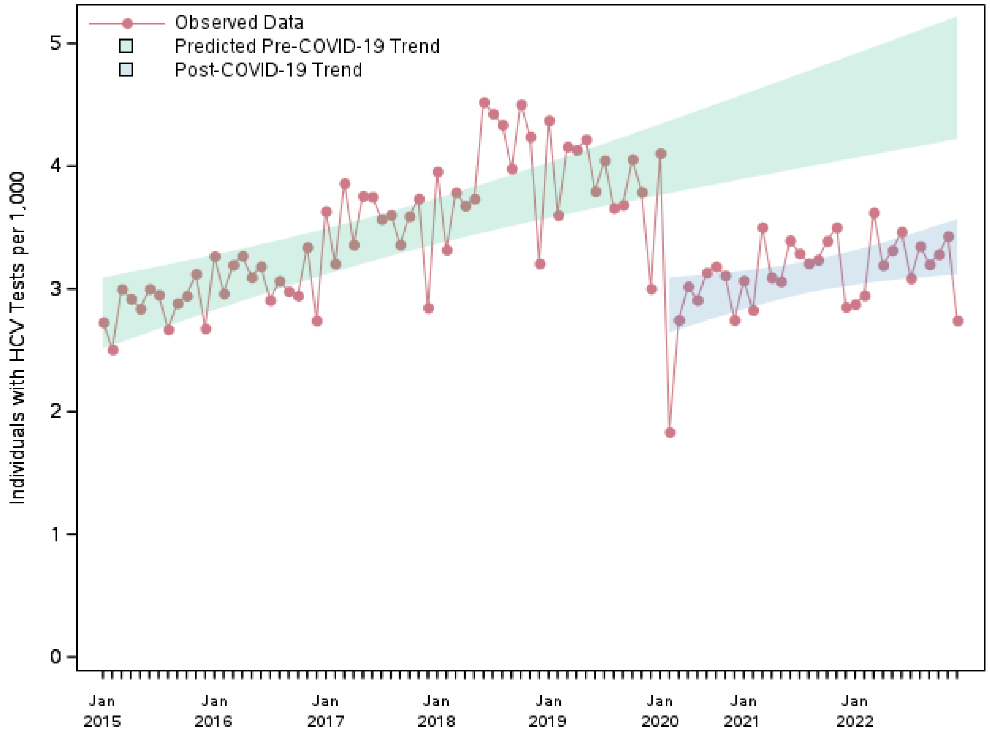

Figure S5. Observed monthly rate of individuals per 1,000 with AB or RNA tests during pre-pandemic and pandemic periods, and the projected counterfactual pre-pandemic trend with 95% confidence band stratified by birth cohort. AB: antibody; HCV: hepatitis C virus; RNA: ribonucleic acid.

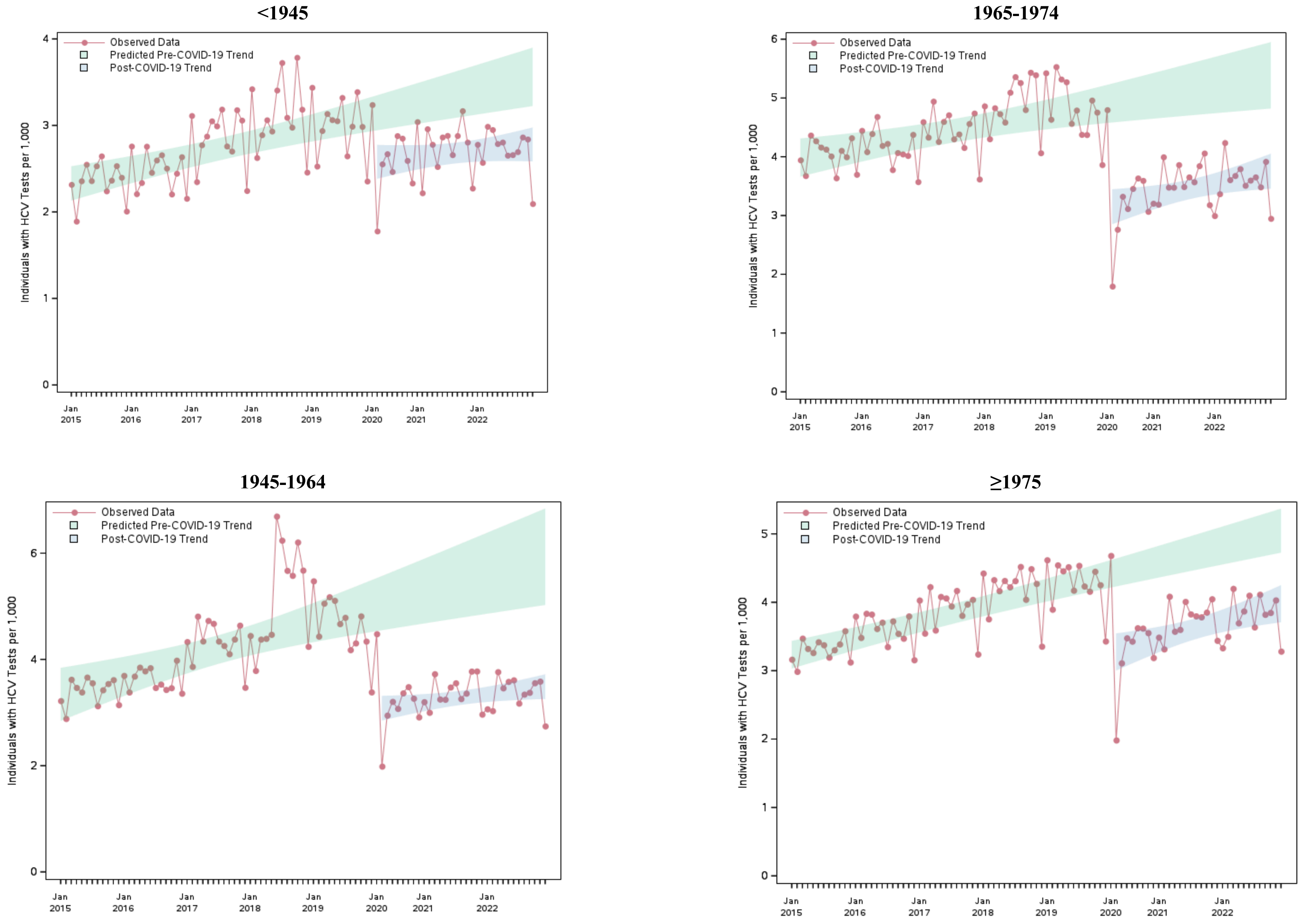

Figure S6. Observed monthly rate of individuals per 1,000 with AB or RNA tests during pre-pandemic and pandemic periods, and the projected counterfactual pre-pandemic trend with 95% confidence band stratified by HIV status. AB: antibody; HCV: hepatitis C virus; HIV: human immunodeficiency virus; RNA: ribonucleic acid.

HIV positive

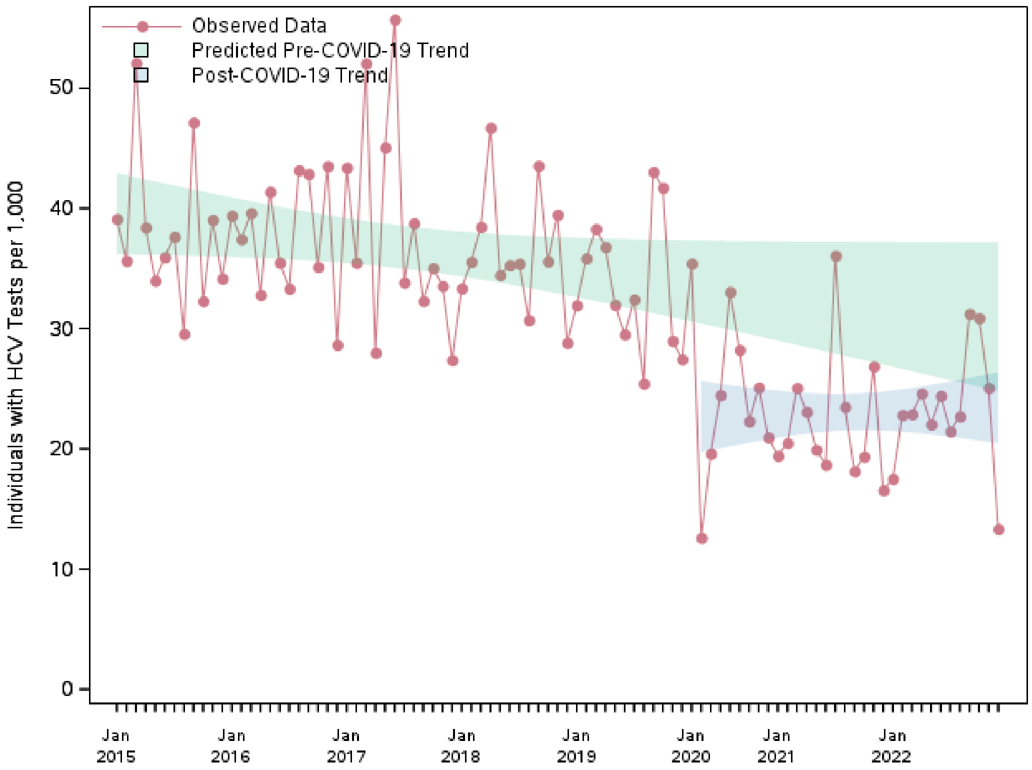

HIV negative

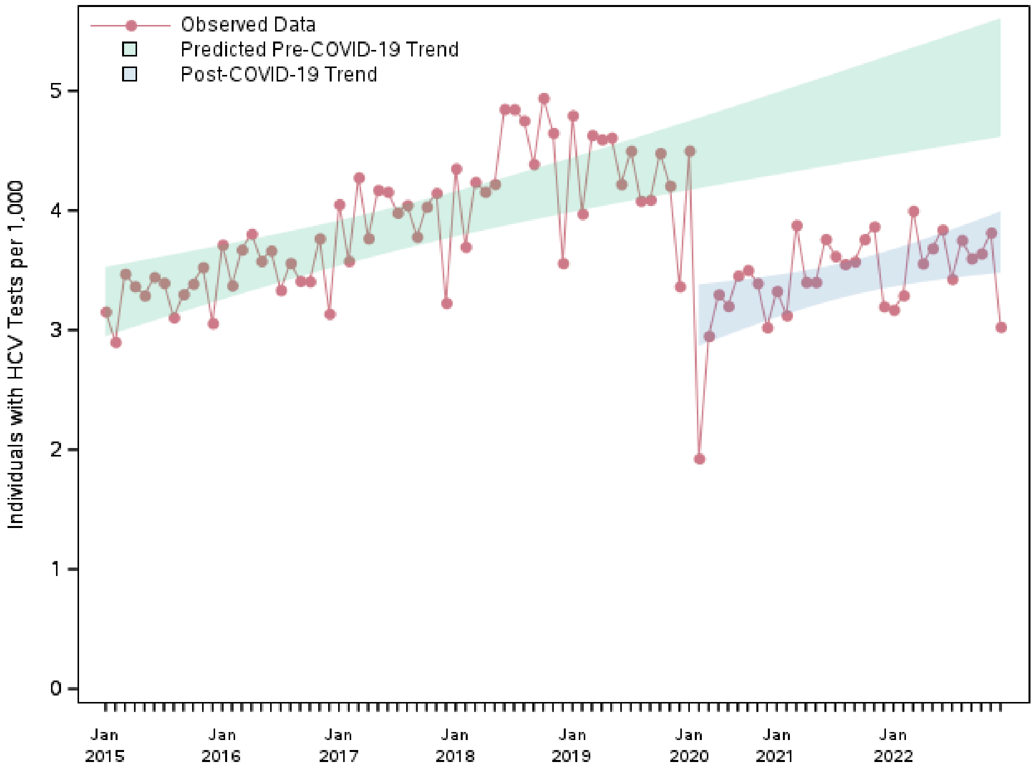

Figure S7. Observed monthly rate of individuals per 1,000 with AB or RNA tests during pre-pandemic and pandemic periods, and the projected counterfactual pre-pandemic trend with 95% confidence band stratified by homelessness status. AB: antibody; HCV: hepatitis C virus; RNA: ribonucleic acid .

Homelessness: YES

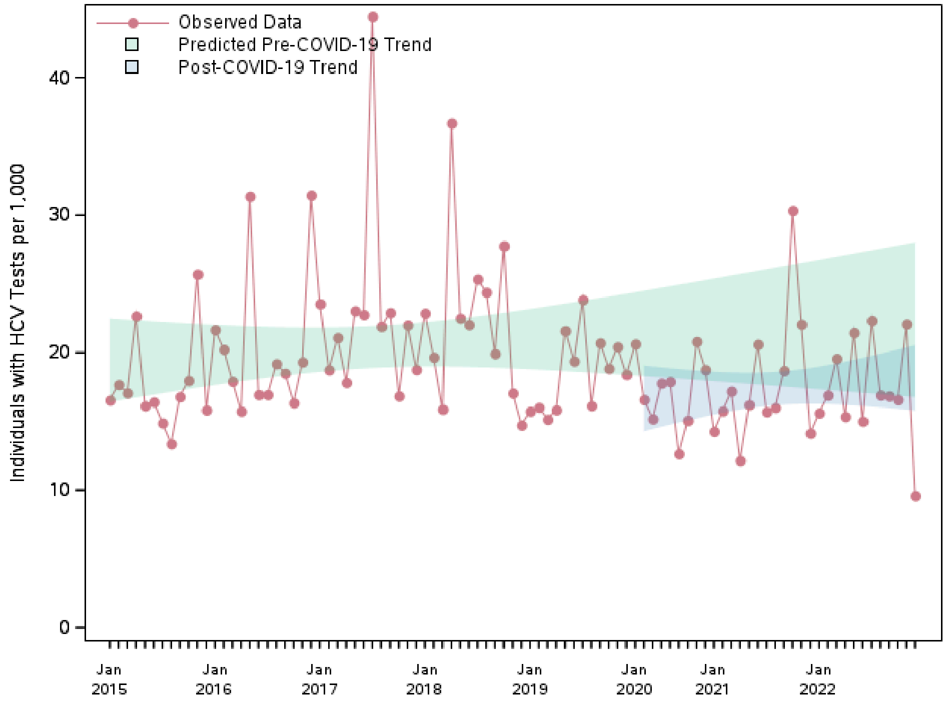

Homelessness: NO

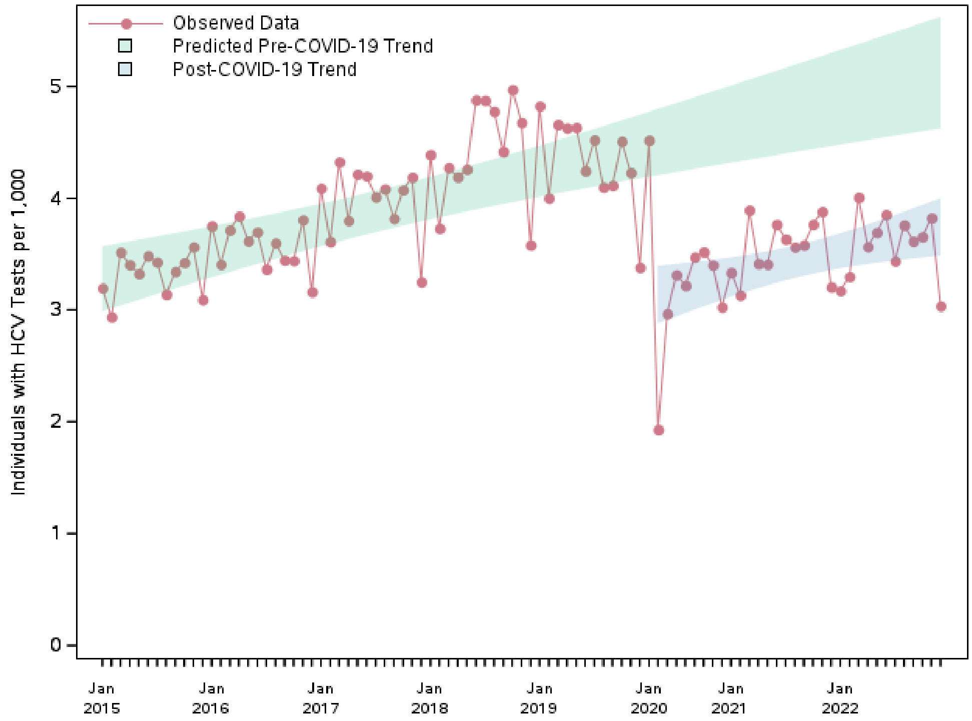

Figure S8. Observed monthly rate of individuals per 1,000 with AB or RNA tests during pre-pandemic and pandemic periods, and the projected counterfactual pre-pandemic trend with 95% confidence band stratified by people who inject drug status. AB: antibody; HCV: hepatitis C virus; PWID: people who inject drugs; RNA: ribonucleic acid.

PWID: YES

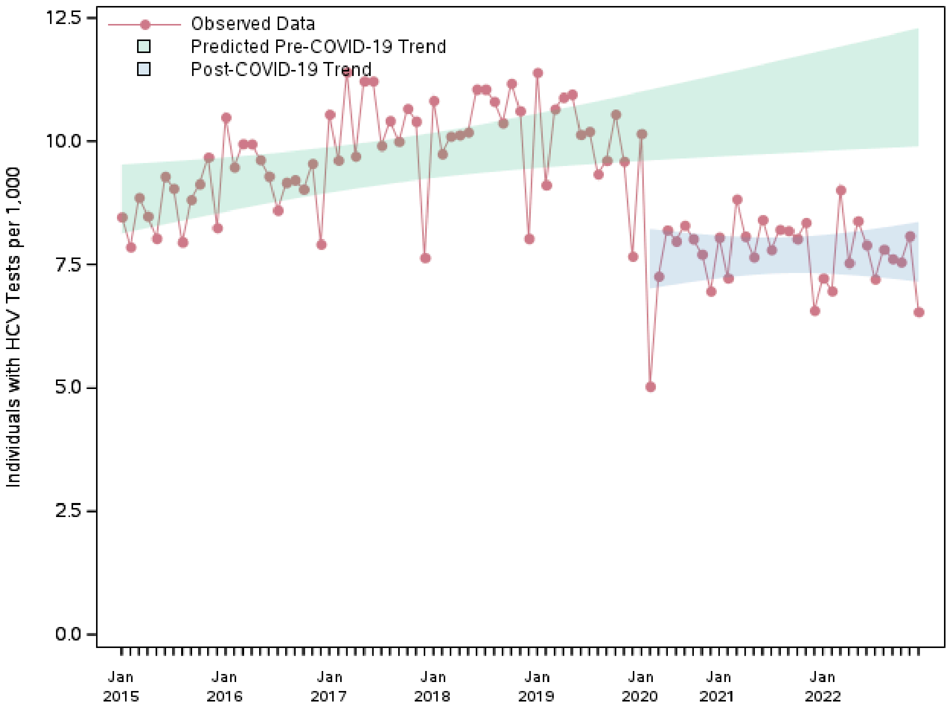

PWID: NO

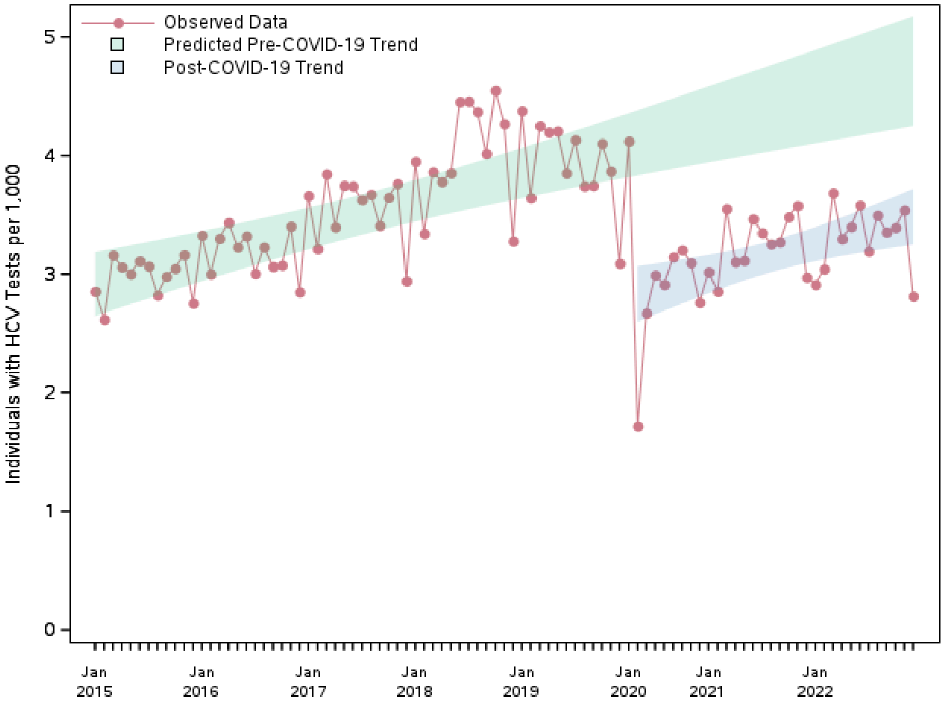

References

1. Richard L, Booth R, Rayner J, Clemens KK, Forchuk C, Shariff SZ. Testing, infection and complication rates of COVID-19 among people with a recent history of homelessness in Ontario, Canada: a retrospective cohort study. CMAJ Open. 2021 Jan-Mar;9(1):E1-E9.
2. Richard L, Carter B, Nisenbaum R, Liu M, Hwang SW. Identification of homelessness using health administrative data in Ontario, Canada following a national coding mandate: a validation study. J Clin Epidemiol. 2024 Aug;172:111430.
3. MHASEF Research Team. Mental health and addictions system performance in Ontario: a baseline scorecard. Toronto: ICES; 2018. Available: https://www.ices.on.ca/Publications/Atlases-and-Reports/2018/MHASEF Accessed on February 14, 2025.
4. Lapointe-Shaw L, Georgie F, Carlone D, Cerocchi O, Chung H, Dewit Y, et al. Identifying cirrhosis, decompensated cirrhosis and hepatocellular carcinoma in health administrative data: A validation study. PLoS One. 2018;13(8):e0201120.
